# Technology-Aided Assessment of Sensorimotor Function in Early Infancy

**DOI:** 10.3389/fneur.2014.00197

**Published:** 2014-10-01

**Authors:** Alessandro G. Allievi, Tomoki Arichi, Anne L. Gordon, Etienne Burdet

**Affiliations:** ^1^Human Robotics Group, Department of Bioengineering, Imperial College London, London, UK; ^2^Department of Perinatal Imaging and Health, King’s College London, London, UK; ^3^Paediatric Neurosciences, Evelina London Children’s Hospital, Guy’s and St Thomas’ NHS Trust, London, UK; ^4^Institute of Psychiatry, Psychology and Neuroscience, Kings College London, London, UK

**Keywords:** cerebral palsy, motor assessment, developmental assessment, robotic-assisted assessment, MRI, functional MRI

## Abstract

There is a pressing need for new techniques capable of providing accurate information about sensorimotor function during the first 2 years of childhood. Here, we review current clinical methods and challenges for assessing motor function in early infancy, and discuss the potential benefits of applying technology-assisted methods. We also describe how the use of these tools with neuroimaging, and in particular functional magnetic resonance imaging (fMRI), can shed new light on the intra-cerebral processes underlying neurodevelopmental impairment. This knowledge is of particular relevance in the early infant brain, which has an increased capacity for compensatory neural plasticity. Such tools could bring a wealth of knowledge about the underlying pathophysiological processes of diseases such as cerebral palsy; act as biomarkers to monitor the effects of possible therapeutic interventions; and provide clinicians with much needed early diagnostic information.

The first 2 years of human childhood are a crucial period for the establishment of the key neural circuits, which subserve the development of normal motor function in humans. Although the corticospinal tracts project into the spinal cord relatively early during *in utero* life (approximately 24 gestational weeks), the final pattern of life-long connectivity is established during the first few post-natal years, through activity-dependent mechanisms, which influence the critical balance between the projection and withdrawal of axons (1). An injury to the developing brain at this critical juncture can result in cerebral palsy, which collectively describes the resulting motor disorder consisting of impairments of posture and movement control (2). While the pathophysiology of motor dysfunction is complex and multi-factorial, recent advances in non-invasive imaging now allow the cause of the majority of cases to be identified as perinatally acquired brain lesions such as those seen following perinatal asphyxia, hemorrhagic or ischemic stroke, and preterm birth (3). Although the resultant motor impairments can be managed (and in some cases improved) with a variety of targeted therapies, the condition remains non-curable and most importantly, there appear to be no bio-physiological mechanisms in place for spontaneous healing or recovery. This apparent failure is perhaps surprising given that neural plasticity is generally considered to be at its most mouldable state during early infancy, suggesting that there should be an increased potential at this stage of life to compensate for neural damage via the retention/formation of alternative axonal routes and synaptic connections (4–11). Early diagnosis of cerebral palsy thus becomes fundamental as it would allow the early identification of appropriate candidates for interventional strategies and also ensure a more efficient allocation of long-term healthcare resources and social support.

In this review, we will describe how novel technology-assisted assessment solutions have the potential to integrate and expand on currently available clinical assessment tools of sensorimotor function, and may provide a sensitive and accurate means of diagnosing cerebral palsy within the first 18 months of post-natal life. Furthermore, we will discuss how these can provide vital new information about the underlying pathophysiology of the disease; thereby also improving our understanding about the mechanisms of and the factors which influence neural recovery (and potential plasticity) following brain injury. The quantitative information potentially provided can also be used to directly monitor the effects of therapeutic intervention both in clinical practice and in the research setting, and in the case of imaging, visualize their effects. To put the techniques in context, we first briefly review currently used clinical assessment tools, and then the small body of studies, which describe using “technology-assisted” tools to obtain objective measurements of how very young children interact with toys fitted with movement sensors. We consider the predictive utility of Magnetic Resonance Imaging (MRI) assessment and discuss how novel task-based functional magnetic resonance imaging (fMRI) paradigms using automated robotic devices can additionally assess the functional state of the neonatal brain. Finally, despite the increasing use of such tools in adult post-stroke assessment and fMRI experiments, their use with children has been limited. We will therefore also discuss the relative benefits and draw-backs to these approaches, and the challenges inherent to developing technology-assisted devices so that they are suitable for the young infant population.

## Early Infant Motor Development and Assessment

Within the first few post-natal months, infants rapidly acquire new patterns of posture, muscle tone, and motor behavior, with spontaneous but seemingly non-goal-orientated movements replaced by an increasing repertoire of purposeful goal-directed movements (12). This change allows the developing infant to interact in an increasingly active manner with its surrounding environment: thus allowing exploration, early learning, communication, as well as maintaining musculoskeletal integrity (13). The ongoing ontogeny of particular motor skills during early infancy is sufficiently systematic that milestones representative of their age of attainment (such as standing and walking independently) can be generally used as relatively robust markers of gross motor development. The first clinical suggestion of developing cerebral palsy is therefore often a delay in milestone attainment, or an observable deviation from typical motor behavior (such as asymmetrical hand use), both of which may not be evident until well after 12 months of age. Making a diagnosis of cerebral palsy earlier in infancy is complicated not only by the rapidly evolving and dynamic nature of early human development, but also by the relatively restricted repertoire of abilities in infants in terms of motor, cognitive, social, and communication responses on testing. Of crucial importance, however, specifically developed clinical assessment tools have found that subtle patterns of neurological abnormality can be identified from a very early age, thus highlighting the feasibility and validity of early diagnosis.

### Current clinical assessment tools

Clinical assessment tools typically involve a single or multiple assessors (usually health care professionals who have been specially trained to administer the test) observing or interacting with an infant, and subsequently scoring the motor behavior or performance in particular tests. Scoring is generally done using ordinal scales, which may be dichotomized (i.e., yes/no reflecting an infant’s ability to perform a task), or have a range of values, which reflect performance with reference to a pre-defined rank order (relative to a population appropriate spectrum of performance). Recent systematic reviews have identified neurobehavioral and neuromotor assessments suitable for use in infants and evaluated their validity and reliability at: (i) discriminating between individuals who are/are not affected by neurological or motor dysfunction, functional limitations, or disabilities at the time of assessment (discriminative ability), (ii) predicting future neuromotor performance, condition, or outcome based upon performance at the time of assessment (predictive ability), and (iii) evaluating longitudinal changes in neuromotor performance, and the impact of intervention (evaluative ability) (14, 15).

Due to the current lack of a criterion standard for neonatal assessment, all available tools are criterion referenced, and no individual tool offers the best possible discriminative, (long and short term) predictive, and evaluative properties (14). While it is proposed that combining assessments that measure different constructs may yield the best psychometric results, this approach is often impractical. A suitable tool should thus be chosen by considering the primary purpose of the assessment (14). In general, due to the statistical effects posed by the numerous environmental and developmental confounds, evaluative validity of current assessment tools has been sparsely and poorly reported (16). Of the identified assessment tools, the Test for Infant Motor Performance (TIMP) was found to be a good all-round tool (and the only suitable evaluative assessment tool), and the assessment of General Movements (GMs) to be the best long-term predictive assessment tool for the given age range (14).

The TIMP was specifically designed for the assessment of infants between 34 weeks post-menstrual age (PMA) and 4 months post-term, and consists of two scales for rating both spontaneous motor behavior and motor responses to stimulation (17). Spontaneous motor behavior is scored by 28 observed items consisting of movements such as head centering, reaching, and finger movements, while the elicited motor behavior is scored by performance in 31 items, which assess the infants response to placement, handling, and visual or auditory stimulation (17). Of particular importance, specific items in the TIMP have been formally assessed for their utility in the prediction of cerebral palsy, and have been found to correlate well with developmental outcome at 6 months of age as assessed by the Bayley Scales of Infant Development (18, 19). However, while the TIMP has been found to have excellent sensitivity (with over 90% of infants correctly predicted to develop cerebral palsy) and good specificity (with 76% of infants correctly predicted not to develop later cerebral palsy) for the prediction of motor outcome at 12 months of age, approximately 35 min are required for an experienced practitioner to administer the test, and its predictive validity has been found to differ depending on the age of testing (20, 21).

It has been suggested that the assessment of GMs may provide the most objective measurement of an infant’s clinical status as it performed using a video recording of their spontaneous movements; thereby eliminating the need for patient handling and minimizing inter-rater variability (22, 23). The fundamental premise of GM assessment is that the quality and quantity of self-generated motor behavior is an accurate representation of the condition of the developing nervous fetal or infant nervous system (22). GM assessment within the first 4 months of life has been found to predict cerebral palsy at 2 years of age with an excellent sensitivity (>90%), and a good, but variable specificity (between 60 and 100%).

Given the previously discussed implications of activity-dependent processes on the nervous system development, a patient group of particular interest is young infants at high risk of developing unilateral cerebral palsy, in whom an accurate measure of early bimanual function may enable the development of interventions using the apparent window of enhanced neuroplasticity in this period. Taking this into account, the mini-Assisting Hand Assessment (AHA) tool for infants between 8 and 18 months of age at high risk of unilateral cerebral palsy is currently under development (24, 25). The aim of the mini-AHA is to transpose the established AHA clinical assessment for older children to this younger population, whilst maintaining the tool’s capacity to accurately assess a child’s ability to use each of their hands independently, and additionally to incorporate more cognitively demanding (and perhaps more discriminative) bimanual tasks (25). Much like the AHA, the mini-AHA proposes to assess, discriminate, and evaluate longitudinal changes in the usage and performance of the affected hand in children with unilateral cerebral palsy. This is done by observing the children play with a series of specifically designed toys, and scoring their ability (on a four point scale) to perform twenty increasingly difficult object-related manipulative tasks, ranging from simple holding of an item to bimanual toy interaction (24). A preliminary internal-scale validation study has found that mini-AHA items can be ordered hierarchically using a Rasch model fit, such that a discrete monotonic increase is seen in both the item difficulty coefficient and the infant’s ability to use their affected hand (24). Children were very finely separated according to their level of ability, and, crucially, test scores were found not to be affected by age.

The mini-AHA and in general all observation-based clinical assessment tools however, suffer from several limitations. Firstly, longitudinal studies on large population cohorts are required to validate the discriminative and predictive power of the tool. Before such tools can be made common place in clinical practice, all of the administering health care professionals must undergo extensive (and expensive) training sessions to minimize the effects that subjectivity and resultant inter-rater variability may have on the final score. As the majority of clinical assessment tools are scored using categorical or ordinal scales, they all suffer from limited resolution, affecting their ability to provide new information about the disease mechanisms and natural history. Finally, the assessment procedures are often extremely time-consuming, resulting in prolonged and costly commitments of clinical staff, and facilities.

### Sensor-based, automatic assessment tools

Here, we define a “technology-assisted assessment tool” as an object or device, which has been specifically designed to automatically induce and/or precisely measure movement. In this context, an “intelligent” or “instrumented” device has been fitted with highly accurate sensors for measuring detailed aspects of motor behavior. A “robot” is equipped, in addition to sensing components, with motors for inducing controlled patterns of movement. An obvious benefit of such tools is that they provide quantitative measures of complex facets of motor function, which can then be used for both intra-subject (longitudinal) and inter-subject (both cross-sectional and longitudinal) comparison (26, 27). This could potentially allow statically robust hypothesis testing for clinical trials of novel therapeutic strategies, thereby greatly improving study power, and reducing the number of subjects required to identify a significant effect (27). Further significant advantages include removing the subjective element from any assessment, and that they can be designed to be both simple to use and time efficient, thus saving valuable clinical time and training (26).

Rather than scoring motor behavior to a relatively inflexible ordinal scale of values, the data type provided by technology-assisted devices is usually on a continuous ratio scale, thereby offering far greater resolution and potentially an important means with which to gain new insights about pathophysiology (27). Furthermore, while traditional clinical assessment provides information about aspects of motor function and behavior which can only be directly observed and felt by an examiner, technology-assisted tools can provide detailed measurements of multiple facets of motor function including those which cannot be perceived through a human observer. These include kinematic information (about the temporal and spatial quality of movement), kinetic information (about the force and work associated with motor function), and neuromechanical information (about musculoskeletal dynamics and feedback via information about impedance and viscoelastic properties) (27). Light-weight sensors and robot-assisted assessment tasks can also be designed around everyday objects and motor behavior (such as turning a door handle or pouring a drink). The derived data can then be used to calculate physiologically meaningful measures of motor behavior such as the active range of motion, target error, movement smoothness, movement time, movement deviation, and force direction error [for review see Ref. (27)].

**Figure 1:**
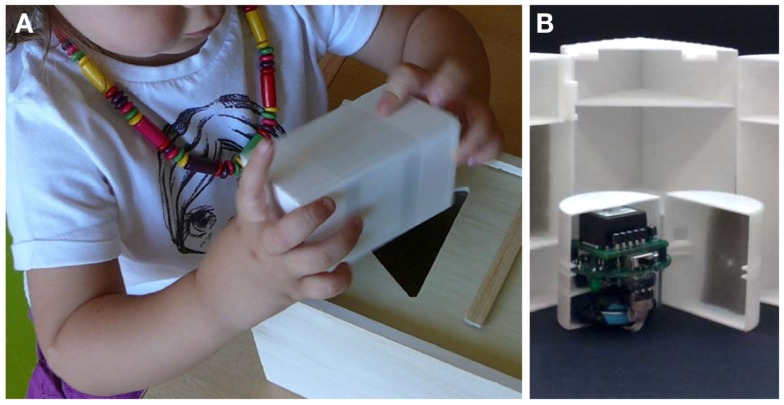
**Instrumented sorting block toy**. The traditional sorting block is a relatively complex toy for young children, which ultimately requires good spatial orientation, grasp control, force control, and motion planning. In this instrumented version of the toy, the block has been equipped with an inertial measuring unit **(B)** to track and record the object’s orientation and linear accelerations during grasping, manipulation, and reaching tasks **(A)**.

While there is increasing evidence that robot-assisted assessment tools can complement or in some cases replace standard clinical tools in adult patients (e.g., stroke survivors), there have been very few studies which have implemented the approach to evaluate motor function in early infancy. Designing such a tool is clearly challenging as it must be non-threatening to the infant, interesting enough to encourage meaningful interaction, unobtrusive (and light-weight) enough to allow natural motor behavior but strong enough to withstand potentially rough play (Figure 1) (28). With this in mind, another direction consists of instrumenting toys with sensors, enabling assessment as well as a game-like interface, similar to rehabilitation robots (though without the capability to move a child’s limbs). Early studies summarized in Table 1 have created such “intelligent toys” from familiar toys equipped with light-weight sensors (28–31), that encourage play through goal-orientated activity with regular positive feedback (29, 32–35). In general, these studies have demonstrated only the feasibility of intelligent toys to derive quantitative movement measures in healthy young infants. An instrumented sorting block toy was shown to be sufficiently sensitive to demonstrate differences in performance related to motor planning and task difficulty (31). This concept has also been extended further for babies by integrating intelligent toys into a baby gym, providing an engaging and involving enriched environment for an ecological assessment of reaching patterns and grasping forces in infants aged between 4 and 9 months (32, 34, 35). Longitudinal trials have demonstrated that this system is capable of providing quantitative measures of power grip maturation patterns in healthy infant subjects, and has the potential to be employed to objectively assess hand function in this naturally uncooperative population (34, 36). Meaningful measures about standing balance and gait in older children have also been derived from commercial gaming devices such as the Nintendo Wii, with the advantage that the device is generally familiar and attractive to the children being assessed (37).

**Table 1:** **Summary of studies that have developed instrumented toys to quantitatively assess movement in young children**.

Study	Design	Age group	Measures	Findings
Campolo et al. 2008 (28)	Instrumented ball toy sensorized with inertial units (accelerometer, magnetometer, and gyroscope) and custom-made force sensors (0–20 N)	6 months and above, intended for children suffering from autistic spectrum disorders	Applied force Spatial orientation and acceleration of object movement	Not formally tested with infant subjects
Cecchi et al. 2008 (29)	Instrumented rattle, sensorized with inertial units (accelerometer, magnetometer, and gyroscope) and binary contact sensors	9 months and above	Grip shape Spatial orientation and acceleration of movements	Preliminary test with three infants (24 months old) showed typical 3–4 finger grasp patterns
Cecchi et al. 2010 (34) Cecchi et al. 2010 (35) Serio et al. 2011 (32) Sgandurra et al. 2012 (36)	“Biomechatronic gym” (instrumented baby play gym) consisting of three toys (cow-toy, flower-toy, and ring-toy) integrated with visual and auditory stimuli. Toys contain piezo-resistive pressure sensors (0–5 psi) and force sensing resistors (0–20 N)	4–9 months old	Palmar (power) and precision grasp: applied pressure and force range Distinction between lateralized or centralized activity defined by position of toy during play with respect to midline	Tested longitudinally with seven infants: Central tasks: trend toward decreasing bimanual activity (and increasing unimanual activity) with increasing age for central tasks
				Lateral tasks: significant increase in contralateral action with increasing age
				Increase in occurrence of precision grasp and reduction in occurrence of power grasp with increasing age. Force applied during both grasp types increases with age
Klein et al. 2011 (30)	Instrumented block sorting toy, sensorized with force sensors, and infra-red proximity sensors	Age range not specified	Applied force on object lid as a function of shape and location	Tested with nine blind-folded healthy adult volunteers, showed significant performance improvement with learning
			Correct insertion of object, task completion time, number of mistrials, and percentage of time spent far from the target	
Campolo et al. 2012 (31)	Instrumented block-box toy, sensorized with magneto-inertial sensors	12–36 months old	Tracking orientation during object placement	Tested with four healthy infants (14–25 months old) for acceptability
			Vertical and horizontal alignment errors and insertion time	
Serio et al. 2012 (33)	Commercially bought horseshoe-shaped toy, sensorized with silicon chamber for pressure measurement (0–5 psi)	4–9 months old	Bimanual applied pressure during power grasp	Not formally tested with infant subjects

While studies in adult stroke patients and early feasibility work in infants of technology-assisted assessment are clearly promising, there are currently no accepted standards for their implementation in clinical practice, and the majority of tools and their derived measures have not been validated or systematically evaluated (27). If the field is to be advanced, there is therefore a need to ensure that they can be optimized to collect clinically relevant information, which can be clearly related to the underlying neurophysiology. Moreover, if robot-assisted tools are to be made widely available and regularly used in clinical practice, it is also vital that their ease-of-use and cost are also carefully considered during the design.

### Magnetic resonance imaging assessment

In recent years, non-invasive imaging techniques have dramatically transformed both clinical practice and neuroscience by providing a detailed means with which to visualize the causative pathology of conditions such as cerebral palsy and study the underlying disease mechanisms. Commonly used tools such as cranial ultrasound (CrUSS) can provide invaluable bed-side images of the brain during the neonatal period, thus providing clinicians with an important means with which to identify acute pathology and general anatomy. MRI scanning allows the acquisition of high spatial resolution images with excellent tissue contrast, which can be obtained in any plane (including in three dimensions), and without the risks of ionizing radiation. In addition, MRI can provide detailed visualization of the whole brain (including inferior areas such as the cerebellum, which are often poorly seen on ultrasound). This can therefore allow the precise delineation of pathology (such as hemorrhage or infarction) and its associated effects on brain structure. Moreover, the information acquired about each volume of tissue (known as a “voxel”) acquired within an MRI scan is in the form of a quantifiable signal, making it highly amenable to mathematical modeling techniques and statistics. However, MRI scanning is expensive; requires for specialist staff and facilities; and the image acquisition itself is noisy and particularly susceptible to image artifacts generated by movement. Despite these draw-backs, MRI is becoming increasingly common in the clinical setting; and has established a clear place in medical and neuroscientific research due to its inherent flexibility, which allows the detailed visualization and measurement of diverse aspects of brain tissue structure, composition, and function.

### Functional MRI and robotic stimulation devices

Through the measurement of temporal changes in the Blood Oxygen Level Dependent (BOLD) signal, fMRI can measure and spatially map brain activity with relatively good spatial resolution (in the millimeter range) and fair temporal resolution (usually a few seconds) (38, 39). A typical fMRI experiment consists of intermittently presenting a subject with an external stimulus (or asking them to perform a task) while a series of whole brain images are rapidly acquired; and localized areas in the brain are then identified in a subsequent (usually off-line) statistical analysis where the BOLD signal has significantly changed from baseline in a manner corresponding to the timing of the stimulus or task (40). An optimal fMRI experimental paradigm would therefore be capable of inducing a robust and repeatable change in the sampled BOLD signal in discrete regions of the brain, and at a frequency, which is clearly distinguishable from noise (40, 41). Although fMRI is now widely used in neuroscience and psychology experiments to map and characterize stimulation-induced functional activity across the whole brain, there have been relatively few systematic studies which have applied the technique to study functional activity in the developing infant brain [reviewed in Ref. (42–44)]. The majority of these studies have reported functional responses to visual and auditory stimulation (45–47), and a smaller number to tactile stimulation (48, 49). It is also of note that many of these studies used stimulus presentation methods, which were not specifically designed for infant subjects and/or were manually controlled resulting in an inconsistent pattern of stimulation.

A critical advantage of a fully automated robotic stimulus system for fMRI experiments is that it can provide a truly consistent pattern of stimulation, including precise control of the time of stimulus onset, amplitude, and frequency (50, 51). Of vital importance however, such a system must be MR-safe (i.e., poses no physical risks in all MR environments) as defined by the American Society and Material and the U.S. Food and Drug Administration (US-FDA) (http://enterprise.astm.org/) (50). In addition, all device components, which are placed inside the examination room and independently generate radio-frequency waves must be contained within their own electrically conductive shielding (known as a Faraday cage) to prevent electromagnetic interference during image acquisition (52) and the functioning of the motors should also not be disturbed by the very large magnetic field produced by the scanner. Devices should be made from non-ferromagnetic materials, and may require non-standard engineering solutions to carry out the desired action (such as using pneumatic, hydrostatic, or cable transmission) and sensing (such as fiber-optic transmission) to keep all potential interferences outside the scanner room (50), or suitable and tested shielding (53). The design of the device must also take into account the relatively small space available inside the bore of the MRI scanner, and the position of the subject (usually lying down supine). To ensure that all of the aforementioned issues are appropriately considered, the development of MRI/fMRI compatible robotic devices often requires flexible solutions and/or compromise and should involve close collaboration between engineers, MR physicists, neuroscientists, and clinicians (see Table 2).

**Table 2:** **Suggested requirements for an fMRI compatible robotic device**.

Contain no ferrous materials and be fully MRI/fMRI compatible
Be mechanically safe to avoid distress or possible harm to the infant
Be able to provide stimulation synchronized with fMRI acquisition
Be able to induce stimulation patterns at a controlled amplitude and frequency capable of eliciting robust functional responses
Be possible to monitor the operation of the stimulus remotely to ensure consistent stimulation was occurring and that no potentially harmful events could occur
Be light, small, and flexible enough to avoid the infant suffering movement restriction or discomfort
Not additionally induce head movements and so avoid resulting image artifacts
Be easily cleanable to prevent infection spreading from one infant to another
Be capable of presenting a stimulation type and pattern, which is appropriate for the neurodevelopmental stage of the study population

**Figure 2:**
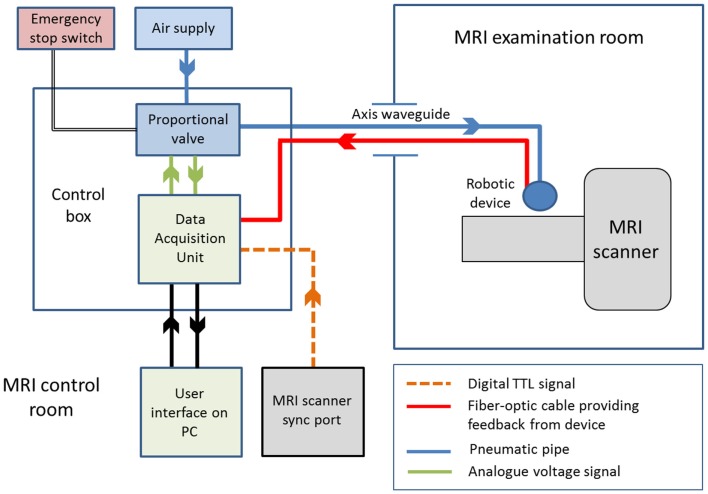
**Schematic diagram of an fMRI compatible robotic device control system**. The robotic device can be controlled remotely via a control box located in the MRI control room. Actuation of the device can be achieved via timed opening of the proportional valve, which allows pressurized air through the pneumatic connection (running through the axis waveguide) to the device in the MRI examination room. Complete control of the system is achieved via a user interface running on PC software, and integration of the multimodal information through a data acquisition unit.

We have recently developed a set of robotic devices that address these challenges, as well as a control system capable of delivering safe and reproducible patterns of stimulation for fMRI experiments with young infant subjects (43, 44, 51, 54). To ensure MRI safety, all of the components within the MR examination room are entirely metal-free, and the devices are actuated by pressurized air through pneumatic tubing and a standard PC situated in the MR scanner control room (schematic of the system is shown in Figure 2) (43). Synchronization of the stimulation onset with image acquisition can be readily achieved by detection of the MR scanner TTL (transistor–transistor logic) pulse via the “sync” or “volume trigger” outlet port on most standard MR scanners.

**Figure 3:**
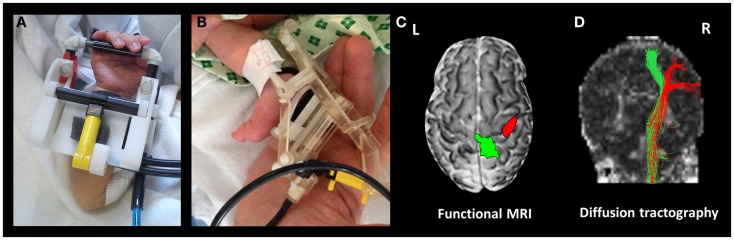
**Functional magnetic resonance imaging compatible devices can be used to precisely map functional activity and axonal pathways**. The devices are fitted to the subjects’ limbs prior to scanning, and can provide a safe and reproducible pattern of stimulation, which is fully automated and synchronized with fMRI data acquisition. Shown are devices fitted to the wrist **(A)** and ankle **(B)**. In a preterm infant at 35 + 4 weeks post-menstrual age, this approach can then be used to identify localized clusters of functional response **(C)** using fMRI (green cluster identified with passive movement of the left ankle, and red following passive movement of the left wrist), and their underlying structural connections can be delineated using diffusion tractography **(D)**.

Using this general system, we have been able to identify well localized and reproducible pattern of positive BOLD brain activity using a variety of stimulation paradigms and fMRI in infants during the preterm period and at term equivalent age (43, 44, 51, 54). The first of these devices was a simple latex balloon, which was placed in the palm of the infant subjects, with timed inflation and deflation resulting in opening and closing of the fingers (43). This allowed us to map brain activity in the primary somatosensory cortices of preterm infants as young as 29 weeks PMA using a block paradigm (43), and to characterize the hemodynamic response to a brief (1 s) stimulus using an event-related paradigm (54). The importance of the preterm period (equivalent to the third trimester of human gestation) was emphasized by the findings of these studies, which demonstrated systematic maturational trends in both the spatial (beginning from a predominately contralateral response in the primary somatosensory cortex in preterm infants to increasing involvement of the association motor areas with increasing age) and temporal characteristics (shorter lag time to the peak of the response with increasing age) of the identified responses (see Figure 3) (43, 54). These initial findings have led to significant refinement of the subject interface to allow an even finer control of the pattern of stimulation, with light-weight robots capable of providing a more specific proprioceptive stimulus with exact and highly reproducible properties to different limbs such as the wrist and ankle (see Figure 3) (51). Furthermore, a fiber-optic position sensor has been incorporated into the robotic interface, thus providing both precise feedback about both the pattern of stimulation and additional information about spontaneous movements made by the subject (51). We were also able to demonstrate the flexibility of the control system by adapting it to allow the presentation of olfactory stimuli, with the odor of formula milk found to induce functional brain activity in the primary olfactory areas of infants at term equivalent age (44).

## Studies Combining Clinical and Technology-Assisted Assessment Approaches

While the majority of studies, which have reported the use of technology-assisted assessment solutions have been promising and have demonstrated their feasibility in children, there have been few which have formally tested their use in the clinical setting. Such work has however been done in the adult post-stroke field, where robot-assisted solutions for assessment and rehabilitation have become increasingly commonplace (27, 55). The validity of objective motor behavioral measures was demonstrated by Bosecker and colleagues (56) who found a high correlation between kinematic and kinetic metrics collected using a robot fitted to the arm, and an extensive battery of standard clinical assessments of upper-limb motor function in a relatively large group (111) of chronic adult stroke patients. Furthermore, robotic tools have been shown to have sufficient sensitivity to detect significant differences in objective measures of arm use (such as task completion time, movement overlap, and phase difference) when performing an everyday task (drinking and pouring) with as few as 10 control subjects and 7 stroke patients (57). A clear difficulty inherent to studies of this population is the heterogeneity of patient groups, which may explain the apparent contradiction of a recent meta-analysis of upper-limb robot-assisted rehabilitation, which found a significant improvement in upper-limb motor function but no clear change in scores of activities of daily living (55).

Of the previously described clinical assessment tools, by nature of its application and off-line scoring, perhaps the most amenable to combination with a technology-based solution for infants is GM assessment. Indeed, studies have shown that the predictive power of GM assessment is preserved even when the assessment is automated by fitting motion tracking sensors to the extremities of the infants (in addition to video recording the infant), and then performing motion feature extraction on a highly filtered motion image using custom computer software (58–60). The sensitivity and specificity of GM assessment for the prediction of cerebral palsy has also been found to be greatly enhanced by combination with MRI at term equivalent age (61).

In comparison to other clinical assessment tools and commonly used neuroimaging modalities (such as CrUSS), MRI at term equivalent age has a relatively good evidence base supporting its use for the prediction of later cerebral palsy (15). It has become the diagnostic investigation of choice in infants who have suffered brain injury at the time of birth, and can provide vital prognostic information through precise delineation of both the acquired lesion and its associated effects. In addition to standard anatomical images (usually T1 and T2 weighted), it is also worthwhile acquiring a diffusion weighted image (DWI), particularly for identifying areas of early ischemia before it can be readily seen on structural images. Following perinatal stroke, MRI has been shown to be highly predictive of later unilateral spastic cerebral palsy (in the side opposite to the brain injury) if abnormal signal is seen in the ipsilesional posterior limb of the internal capsule (PLIC), thalamus, and perirolandic cortex (62); and if pre-Wallerian degeneration is evident on DWI in the contralesional cerebral peduncle (63). Similarly following Hypoxic Ischemic Encephalopathy (HIE), a number of studies have demonstrated that abnormal signal in the basal ganglia and thalami is highly predictive of dyskinetic or athetoid cerebral palsy (64, 65); injury to the basal ganglia, thalami, and brainstem are predictive of feeding and communication difficulties (66), and changes in myelination of the PLIC predictive of adverse motor outcome (67). Of further interest, a meta-analysis of the predictive power of MRI techniques for predicting adverse outcome following HIE found that abnormal deep gray matter lacate/*N*-acetyl-aspartate (NAA) ratio on single proton MRS (magnetic resonance spectroscopy) has the highest pooled sensitivity (68).

The predictive power of MRI may be further enhanced by the application of acquisition sequences, which can also visualize other diverse aspects of brain tissue composition, microstructure, and function. A clear example of this is tractography derived from diffusion MRI data, which utilizes information about the random diffusion of water inside the brain to delineate the major axon fiber bundles within the brain’s white matter (69). Using this approach, it is possible to further increase the sensitivity of MRI for the prediction of later cerebral palsy by identifying subtle asymmetry in the microstructural integrity of the corticospinal tracts in infants with focal brain injury (70, 71). When correlated with neurological or developmental outcome, these measures may therefore provide additional prognostic information, and can also be used as highly accurate cerebral biomarkers for studies of pathological effects and treatments (72).

A key feature of fMRI is that it can visualize functional activity in the whole brain, thereby allowing the mapping of large-scale functional networks including connectivity to physiologically important deep brain structures such as the thalamus, basal ganglia, and cerebellum (73). Moreover, spatial information about the localization of functional brain activity derived from fMRI experiments can be combined with that derived from other analysis techniques such as diffusion MRI tractography, thereby allowing a detailed visualization of the macroscopic framework of both functional and structural connectivity (74). While it may not be feasible to perform the aforementioned fMRI studies in the standard clinical setting, it is likely that the results acquired in specific specialist centers may provide dramatic new insights about the pathophysiological processes underlying the development of later cerebral palsy, which will therefore be of relevance to all working in the field. This notion becomes even more compelling, when it is considered that there have been a number of case reports of children in which dramatic sustained alterations in functional neuroanatomy have been seen following focal brain injury earlier in life (75–77). fMRI and advanced imaging techniques such as tractography may therefore present an accurate means with which to characterize and monitor neural (re)organization and neuroplasticity following brain injury, and furthermore to longitudinally monitor how they may be influenced by activity and therapeutic intervention (78).

Despite its prominent place in current clinical practice, there is however a surprising paucity of clinical studies, which have investigated the early predictive value of structural MRI for later cerebral palsy in other high risk populations (in particular prematurely born infants born without evidence of focal brain pathology) [for recent review see Ref. (79)]. While normal CrUSS has been found to confidently predict a normal motor outcome later in childhood, MRI at term equivalent age has a high predictive sensitivity for cerebral palsy but suffers from poor specificity, especially in infants with moderate cerebral abnormalities (80, 81). This limitation may be due to small study population sizes (and in particular the relatively small number with adverse outcome) leading to wide confidence intervals. It may also represent limitations inherent to the imaging techniques (such as restricted spatial resolution or contrast), the unpredictability of later childhood influences on neurodevelopment, or the lack of longer term diagnostic information (81). With the increasing availability of MRI, the development of MR compatible incubators and specialized physiological monitoring equipment, it will likely become possible to resolve this uncertainty (82, 83).

## Summary

With the aim of guiding and assessing early intervention strategies, an ongoing goal must be to develop accurate assessment tools, which are capable of providing enhanced diagnostic and prognostic power at an earlier developmental stage during infancy. Future assessment tools should not only have an increased specificity and sensitivity for predicting cerebral palsy, but could also be predictive of the severity of later cerebral palsy (rather than just its occurrence). The integration of sensor- and robot-assisted techniques into both clinical and neuroimaging assessment may allow the collection of detailed measures of motor function and development, thereby giving clinicians much needed patient-specific information on their outlook, recovery and therapeutic effectiveness.

## Conflict of Interest Statement

The authors declare that the research was conducted in the absence of any commercial or financial relationships that could be construed as a potential conflict of interest.
